# 660. Pediatric Rotavirus Hospitalization Rates in the Military Health System Before and During the COVID-19 Pandemic

**DOI:** 10.1093/ofid/ofad500.723

**Published:** 2023-11-27

**Authors:** Matthew Penfold, Sarah Prabhakar, Apryl Susi, Michael Rajnik, Cade M Nylund, Matthew Eberly

**Affiliations:** Uniformed Services University of the Health Sciences, Fairfax, Virginia; Uniformed Services University, Bethesda, Maryland; Uniformed Services University, Bethesda, Maryland; Uniformed Services University of the Health Sciences, Fairfax, Virginia; Uniformed Services University of the Health Sciences, Fairfax, Virginia; Uniformed Services University of the Health Sciences, Fairfax, Virginia

## Abstract

**Background:**

Rotavirus, a vaccine preventable disease, causes significant morbidity and mortality in children < 5 years of age. Globally, the COVID-19 pandemic reduced the rates of pediatric vaccinations, and the Military Health System (MHS) was no different (Figure 1). This gap in immunizations left many children vulnerable to preventable illnesses, including rotavirus. We hypothesized that the reduction in rotavirus vaccine administration during the pandemic would lead to an increase in rotavirus related hospitalizations within the MHS.

**Figure:**
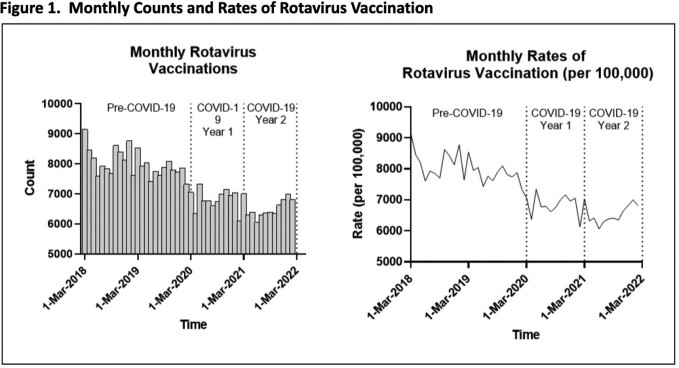

**Methods:**

A repeated monthly cross-sectional study was performed using inpatient data from the MHS to observe differences in rotavirus rates and risk factors before and during the COVID-19 pandemic. Data from March 2018 to February 2020 was considered pre-pandemic, while the following two years (until February 2022), were divided into COVID Year 1 and Year 2. Children included in the study were between 0-59 months of age and those with rotavirus were identified using ICD-10 codes. Poisson regression calculated rate ratios of rotavirus, adjusting for several demographic variables and time period at diagnosis. Monthly counts and rates of rotavirus were also documented.

**Results:**

Across the cohort of 1.14 million MHS beneficiaries, there were 3,503 unique cases of rotavirus infections in hospitalized children < 5 years old (Table 1). During both Years 1 and 2 of COVID-19, there was a significant decrease in rotavirus related hospitalizations compared to the pre-pandemic period (Figure 2). Younger age was associated with increased risk. Patient sex, parent military rank, and region were not statistically significant (Table 2).

**Figure:**
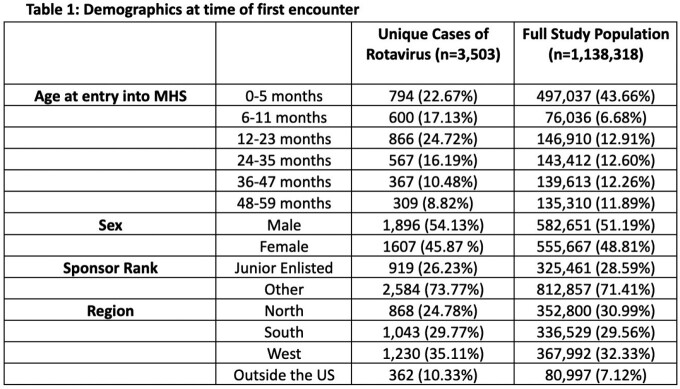

**Figure:**
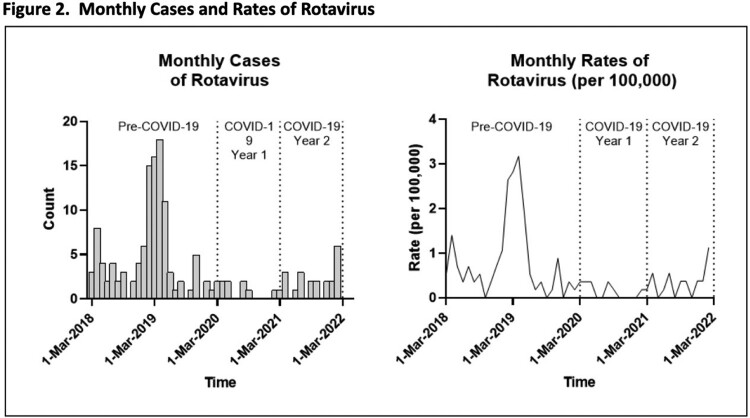

**Figure:**
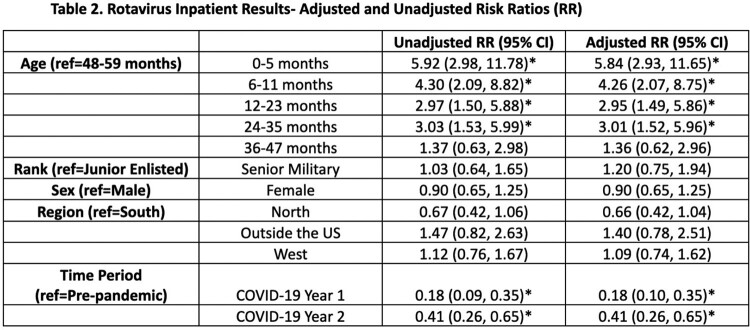

**Conclusion:**

Despite the reduction in routine immunization administration to children in the MHS after the COVID-19 pandemic started, there was not an increase in rotavirus related hospitalizations. In the two years following the start of the pandemic, there was a statistically significant decrease in hospitalizations related to rotavirus in our cohort. Social distancing to include daycare closures, increased hygiene measures, and catch-up immunizations may explain this finding. Continued research into this topic will help determine if rotavirus related hospitalizations return to pre-pandemic levels.

**Disclosures:**

**All Authors**: No reported disclosures

